# Rheological Features and Hereditary Models of Lightweight Sintered Aggregate Concrete Under Cyclic Loading

**DOI:** 10.3390/ma19081539

**Published:** 2026-04-12

**Authors:** Paweł M. Lewiński, Zbigniew Fedorczyk, Przemysław Więch

**Affiliations:** Building Research Institute (ITB), Filtrowa 1, 00-611 Warsaw, Poland; z.fedorczyk@itb.pl (Z.F.); p.wiech@itb.pl (P.W.)

**Keywords:** lightweight sintered aggregate concrete, rheological tests, shrinkage, creep, hereditary creep models

## Abstract

This article compares the analytical results from two models, based on the theory of hereditary creep strain, with experimental results on the rheological properties of lightweight sintered aggregate concrete under cyclically varying loads. In a previous article, the authors analyzed the adequacy of standard models for the same test results. Because the use of standard models is very complex and does not improve the approximation of test results without additional calibration, the authors suggest reconsidering the use of hereditary models for LWAC. The application of four such long-term models was analyzed. Among these models, the Arutiunian theory of hereditary creep with aging and the modified hereditary theory with Bažant aging function yielded quantitatively and qualitatively correct results. The application of hereditary creep theory allowed for the formulation of the total strain as a superposition of strain increments, obtained by an integral equation. This equation was applied to a series of constant stress increments and decrements, as in the case of cyclic loading, and it was mathematically described in segmented form. Knowledge of the properties of LWAC and useful long-term models is essential for the design of prestressed structures made of lightweight aggregate concrete subjected to time-varying loads.

## 1. Introduction

**Figure 1:**
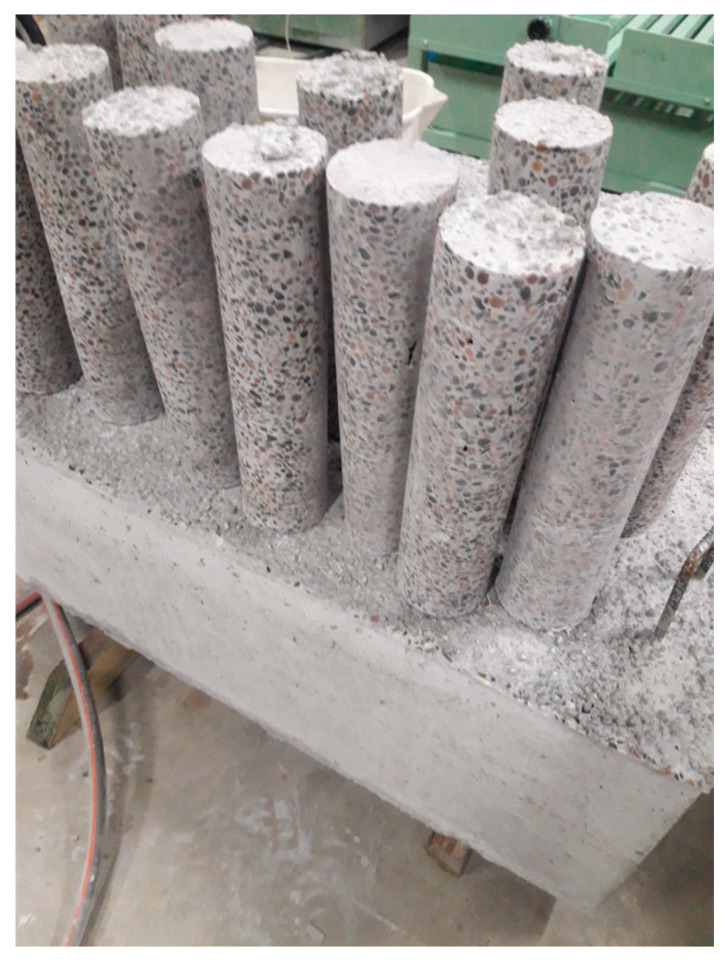
High-LSA concrete cylindrical specimens for elastic modulus, shrinkage and creep testing.

Lightweight concrete with sintered aggregate, also called LSA concrete (Lightweight Sintered Aggregate concrete), as research and engineering practice has demonstrated, can generally be used in concrete structures, including prestressed ones, such as bridges and viaducts, as well as skyscrapers (see Refs. [1,2]). The concrete discussed in this work, but with an improved mix composition, was used in bridge construction. The structural applications of concrete discussed in this article are given in [3,4,5]. This requires meeting certain design criteria that account for the effect of long-term loads, including time-varying loads, on the ultimate limit states of the structure (for example, with respect to prestressing losses) and on the serviceability limit states (for example, with respect to deflections from long-term loads). Accounting for the influence of these long-term effects requires obtaining sufficiently reliable experimental results. The type of lightweight concrete with a relatively new kind of sintered aggregate investigated in the present work was discussed in an earlier publication by the authors [3], which aimed to describe the short-term and rheological properties of this LSA concrete. The lightweight concrete was made with Certyd aggregate (see Figure 1 in paper [3]). This is a relatively new kind of sintered fly ash lightweight aggregate, manufactured since 2015 in Poland [4]. In a subsequent article, the authors addressed the suitability of standard models for the rheological properties of LSA concrete under loads that change cyclically over time [5], which was applied in the next stage of their research, using appropriate equipment, including creep testing machines. This paper describes the final stage of these studies, presenting a comparison of analytical results from two models, based on the theory of hereditary creep strain, with experimental results on the rheological properties of LSA concrete under loads that vary cyclically over time. Research on specific material properties of LWAC with sintered aggregates had begun at the Building Research Institute (ITB, Instytut Techniki Budowlanej) [6,7] earlier, including concretes with the same aggregates that were tested at the Cracow University of Technology (CUT) in the IMiKB laboratory.

The tests performed in the IMiKB laboratory at CUT [8,9,10,11,12,13,14,15,16,17,18,19,20] were conducted on two types of concrete with similar compressive strength values appropriate for prestressed concrete members: LSA concrete and plain concrete, as a reference material. As a result of this widespread research, important papers and monographs on the rheological features of LSA concrete have been published since 2014. One significant monograph [8] presents, among other topics, the state of the art in the field of lightweight structural concrete. Numerous publications have been devoted to the use of LSA concrete for the construction of prestressed floor slabs [9,10,11,12,13,14,15,16,17,18,19,20]. The research conducted in the CUT laboratory has included determining the strength features of concrete, i.e., compressive and tensile strength, elasticity modulus, as well as determining the course of shrinkage and creep strains of concrete. The results of this research prove that the tested concrete has properties suitable for use in prestressed concrete structures. According to the cited works, a low value of creep strain was obtained for LSA concrete. Rodacka investigated the influence of rheological phenomena on the deflection of prestressed lightweight concrete beams [19,20]. The works of Szydłowski et al. [10,11,13,14,15,16,17,18] and Rodacka [19,20] are of fundamental importance for the future design of PC structures made with LSA concrete.

The development of robust, long-term models begins with the Arutiunian theory of hereditary creep strain with aging [21], based on the Boltzmann principle of superposition [22] and the modified Bažant theory of hereditary creep strain with an alternative aging function [23], which have yielded quantitatively and qualitatively correct results. Various ways of generalizing the hereditary creep theory were developed in subsequent works by Bažant and co-authors. The basic modifications mainly concern the compliance function, which plays a basic role in the Volterra integral equation of strain inheritance (see below). Bažant and Osman proposed a double power law for the compliance function [24]. Bažant and Chern developed a logarithmic double power law for concrete creep [25]. These subsequent modifications contributed to improvements in European standards.

Rheological deformations of concrete under loadings changing cyclically over time have been the subject of many scientific articles. Whaley and Neville [26] showed that cyclic loads increase the inelastic strain of concrete compared to static loads. The theoretical relationship describing the development of static creep strain was modified by Hirst and Neville [27] in such a way that the creep deformation under cyclic loads was related to creep strain values under static loads as a function of the stress range and the time of their action. To take into account the results of multi-stage experimental studies and data from the literature, Bažant and Panula applied optimization techniques [28,29], which allowed the development of a practical rheological model (BP). This model was able to reflect creep and shrinkage strains, accounting for the strength of the concrete, age at loading, composition of mixture, environmental conditions, size and shape of samples, etc. This contributed to the development of more precise models adopted in later European standards, as the new models produced more precise results than, e.g., existing ACI or CEB-FIP models. However, the analysis of the research described in this article, in light of the latest standards, was the topic of a separate article by the authors [5]. Ref. [30] investigated the relaxation of five-day cyclic compressive stress at a constant cyclic strain for different frequencies. Bažant and Kim used a semi-empirical rheological model BP [31] to take into account the influence of creep strain (basic, drying, and cyclic), shrinkage strain, and temperature. Creep increase due to the cyclic changes in humidity was formulated in [32], partly on the basis of diffusion theory. An enhanced model of concrete creep and shrinkage in structures (model B3) was devised by Bažant and Murphy [33]. The influence of the scale effect on the results of creep and shrinkage of concrete is discussed in [34].

Ref. [35] presents a semi-empirical model of creep (basic and drying) and shrinkage, taking into account the influence of these strains under cyclic loading conditions on the deflections of RC beams. The study of the behavior of deformed bars in LWAC under cyclically repeated loading in order to determine the bond creep coefficient was the subject of [36]. The theory of cyclic creep of concrete based on the Paris law [37] was developed for structures subjected to repeated variable loads over longer time intervals. The influence of fatigue microcrack growth at subcritical loads was taken into account, and a cyclic creep compliance function with multi-axial generalization was introduced. To determine the effect of loading frequency on the development of strain and elastic modulus, a comparative analysis of concrete creep under compression and cyclic loading was carried out [38] at two given loading frequencies of 0.1 Hz and 1.0 Hz (i.e., high-frequency loading). The analysis showed that at similar average stress levels, cyclic strains were found to be significantly higher than creep strains. A number of important pieces of information in this regard are provided by *fib* MC 2020 [39]. The long-term model presented there, in the form of an integral equation, is based on the theory of hereditary creep strain [21] and on the Boltzmann superposition principle [22].

Many published works, including [40,41,42], analyzed the shrinkage strains of LWAC, including the phenomenon of shrinkage cracks; refs. [43,44,45], also examined stresses resulting from thermal shrinkage caused by the heat of hydration during the setting of this type of concrete, as well as the effect of concrete curing time on the creep of LWAC [46]. The shrinkage and creep strains of LWAC were also the subjects of works [47,48,49,50,51,52,53,54,55,56]. The subject of [57] was the investigation of creep under tension in lightweight aggregate concrete reinforced with steel fibers. The effect of moisture migration on the creep of high-performance and lightweight aggregate concrete was studied by Lopez et al. [58]. An experimental study on the creep and shrinkage behavior of a high-performance ultralight cementitious composite was conducted by Chia et al. [59].

Many studies have examined the effect of variable loads. The influence of stress level during tensile creep has been analyzed in publications, such as [60,61,62,63,64,65,66,67]. The creep–recovery phenomenon has also been addressed in the context of LWAC properties and has been studied in numerous publications [68,69,70,71,72,73,74,75,76,77,78,79,80,81].

Wojewódzki et al. [82] described constitutive models for the relaxation function and generalization of the creep model for triaxial stress state, which have also been recently addressed in *fib* MC 2020 [39]. The influence of various physical parameters on the rheological properties of concrete has been addressed in numerous publications, including monographs by Biliszczuk [83,84]. The fundamental monographs on aspects of concrete creep discussed in this publication are the works of authors such as Neville et al. [85,86,87] and Bažant and Jirásek [88]. Extensive analyses of constitutive models of concrete in the field of shrinkage and creep, including numerical ones, were presented in the monograph by Mitzel [89] and the recently published one by Brunarski [90].

The Building Research Institute has developed a program to investigate the material properties of LSA concrete under long-term load cycles. When starting the research, the authors had two creep testing machines at their disposal, so the tests were carried out using samples made of two mixtures of LSA concrete. Both concrete mixtures were selected for testing based on a literature review, ensuring their representativeness from the perspective of both testing LWAC and its applications in prestressed structures. Based on such criteria, the LSA concrete mixture formulations were adopted as optimized recipes, analogous to those used in the research conducted at the CUT (see Refs. [12,13,16]). According to LSA technology, only two recipes for such mixtures were available; therefore, they were considered representative.

In the Laboratory of Structures, Geotechnics, and Concrete at the ITB, in addition to testing the short-term mechanical properties of the LSA concrete under consideration, with an increased strength class of LC 45/50, research was carried out to determine the shrinkage and creep strains under cyclic loading and unloading [5]. This paper analyzes the course of such strains under variable loading for lightweight concrete samples and two water-cement ratios, similar to a previous publication [5]. In a previous paper [5], the authors investigated the rheological properties of LSA concrete under cyclic loading over time, assessing the suitability of standard models. However, standard models cannot be applied directly and require additional calibration using standard coefficients. These models are very complex compared to models based on the theory of hereditary creep. Because the use of standard models does not improve the approximation of test results without calibration, the authors suggest reconsidering the use of hereditary models for LSA concrete.

The rheological properties of lightweight concrete with relatively new ceramic aggregate sintered from fly ash, when subjected to long-term cyclically varying loads, have not previously been analyzed using the hereditary creep and aging theory of concrete. To fill this gap, the application of four long-term models of the creep and aging of concrete was analyzed. Finally, this paper presents the analytical results of two models, based on this theory, compared with experimental results for the LSA concrete under consideration. The theory of hereditary creep strain allowed the superposition of strain increments for individual loads using an integral equation. This integral equation was applied to a series of constant stress increments and decrements to describe cyclic loading conditions and is presented in a simplified mathematical form.

Compared to previous works, and especially to the authors’ recent publication on the application of standard models [5], the theory of hereditary creep strain with concrete aging, with the proposed approach adapted to cyclic loading using a multi-stage creep model, presented in this article, allows for obtaining the compliance of analytical results with test results in a much less complicated way than in [5], while maintaining comparable modeling accuracy.

## 2. Materials and Methods—Experimental Research Program

### 2.1. Preparation of Specimens of LSA Concrete

The scope of this work concerns the rheological features of two concrete mixtures with lightweight sintered ceramic aggregate (LWAC) subjected to low-frequency cyclic loads. The concrete mixtures prepared for testing were an LC1 mixture with a water–cement ratio of 0.4, and an LC2 mixture with a W/C ratio of 0.5. For comparative purposes, the mixtures were prepared as described in [12,13,16], in accordance with the recommendation of the LSA company, Białystok, Poland, the manufacturer of Certyd, a sintered ceramic aggregate. The concrete mix compositions, based on this recommendation, are given in Table 1, together with test types and the number of specimens. The SKY 686 superplasticizer was used at 3.7 (LC1) and 3.8 (LC2) kg/m^3^, as well as the BV 18 plasticizer at the same rate. Both admixtures are manufactured by Master Builders Solutions Polska, Myślenice, Poland. The density of the fresh concrete mixture was 1960 kg/m^3^ for the LC1 concrete mix and 1980 kg/m^3^ for the LC2 concrete mix. The density of the dried LC1 LSA concrete after 28 days of curing was 1766 kg/m^3^, and 1777 kg/m^3^ for the LC2 LSA concrete, according to [91].

Tall LSA concrete cylindrical specimens for elastic modulus, shrinkage and creep testing are shown in Figure 1.

Tall cylindrical samples of LWAC were collected by core drilling from two blocks made of the LC1 and LC2 LSA concrete mixtures to conduct compressive and tensile strength tests and to investigate the modulus of elasticity, shrinkage and creep strain.

### 2.2. Types of Tests and Samples of LSA Concrete

The LSA concrete under consideration was produced in a concrete plant where the homogeneity of the mixture was ensured by the use of a specialized mixer [3]. The samples were taken from previously poured forms and drilled from concrete blocks in the form of core drillings. After appropriate curing in laboratory conditions, tests were conducted according to the following procedures:

- Secant elasticity modulus of concrete tested on cylindrical specimens, according to [92];
- Compressive strength tested on cube and cylindrical specimens, according to [93];
- Tensile axial strength tested on cylindrical specimens, according to [94];
- Tensile splitting strength tested on cube specimens, according to [95];
- Flexural strength tested on prismatic specimens, according to [96];
- Shrinkage strains tested on prismatic specimens, according to [97], and additionally on cylindrical specimens, according to [98] (to determine creep strains);
- Creep strains tested on cylindrical specimens, according to [94].

The tests were carried out on core hole samples of 94 mm diameter and 190 mm height, to determine the secant elasticity modulus and axial tensile strength, and of 282 mm height for shrinkage, according to [98], and creep, according to [94]. For other tests, the specimens were prepared according to the requirements of the relevant EU codes, as given above. Tests on long-term strains of LWAC induced by low-frequency cyclic loading were the subject of a previous publication by the authors [5], while this paper focuses on comparing the results of rheological model analyses based on the theory of hereditary creep strain, using the results of the above tests. Further details are provided in [3,5].

### 2.3. Testing Machines

**Figure 2:**
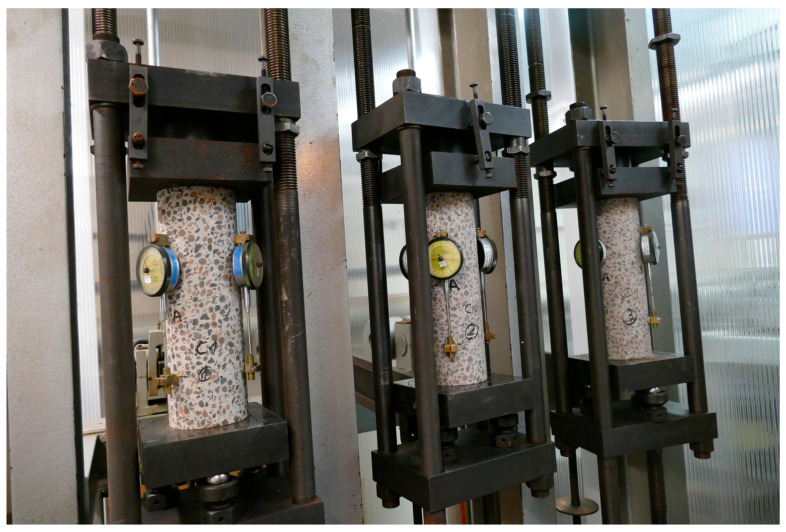
Specimens of the LC1 lightweight concrete in the creep-testing machine.

**Figure 3:**
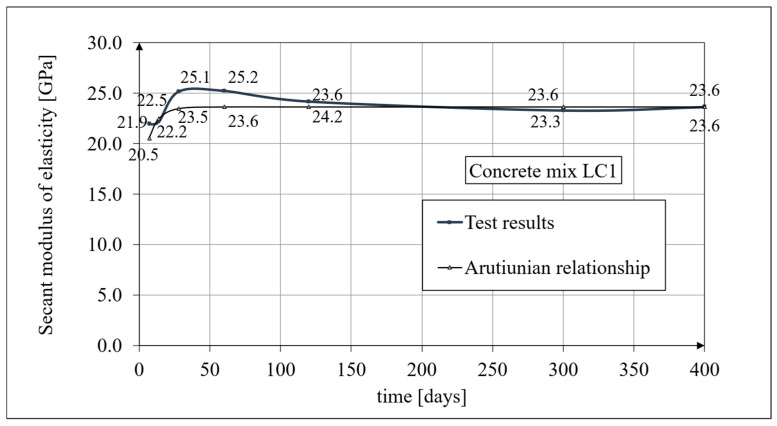
The secant elasticity modulus of the LC1 concrete, according to the tests and the Arutiunian model.

**Figure 4:**
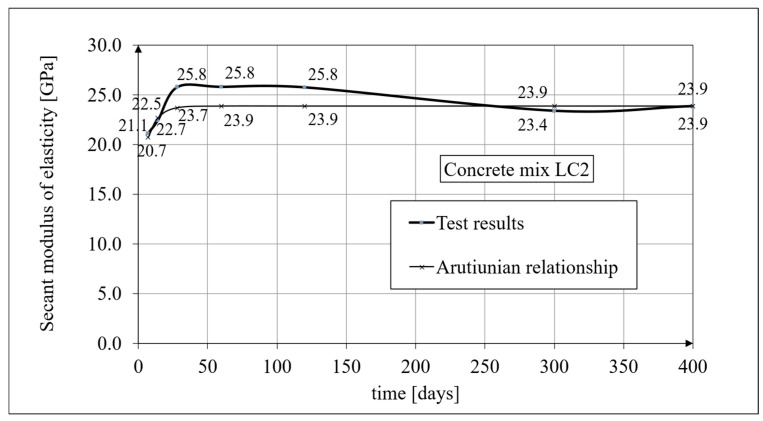
The secant elasticity modulus of the LC2 concrete, according to the tests and the Arutiunian model.

**Figure 5:**
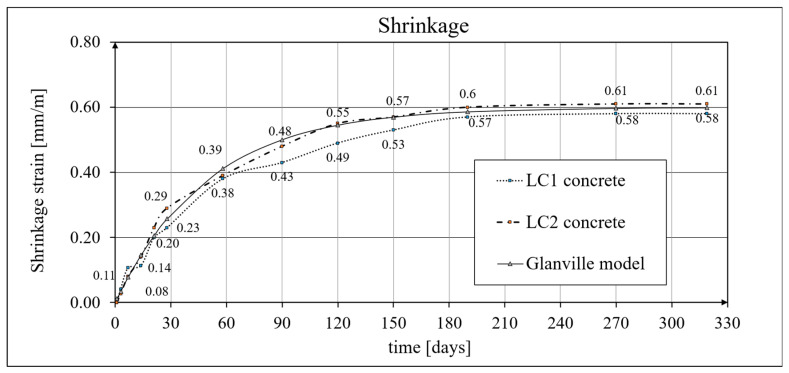
Shrinkage strain, according to Amsler method [97] and Glanville shrinkage model [101].

**Figure 6:**
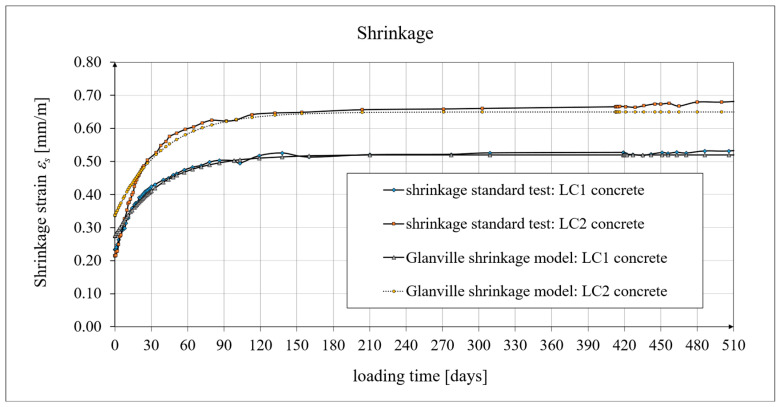
Shrinkage strain, according to standard tests [98] and Glanville model [101].

The equipment used to test the LSA concrete samples is shown in Figures 3–6 in the article [3]. Creep-testing machines (manufactured in former GDR by VEB Werkstoffprüfmaschinen, Leipzig) were used to measure creep strain. Sample testing was conducted according to the requirements of the relevant EU codes [92,93,94,95,96,97,98] and ITB Instruction No. 194/98 [94] (as shown in Figure 2). The testing equipment was of first-class accuracy. More details can be found in the articles [3,5].

### 2.4. General Schedule for Testing Long-Term Strains of LSA Concrete Under Cyclic Loading

Following the research program, basic strength tests on the LSA concrete were conducted as described in [3], while tests on long-term strains induced by low-frequency cyclic loading were carried out according to a special schedule, as described in [5]. Shrinkage tests on the LSA concrete were performed at intervals, according to the reference document [99], until stable results were obtained, and then readings were taken at constant intervals. The tests on the secant elasticity modulus [92] were conducted for a longer period than the basic strength tests. However, the auxiliary shrinkage development tests (according to the new standard [98]) were performed at equal time intervals as the creep strain tests, in accordance with the long-term test schedule. The creep strain tests, according to [94], were carried out in creep-testing machines on six test stands for 1050 days for the LC1 concrete and for 1044 days for the LC2 concrete, covering three loading phases and two unloading phases in both cases, as described below. At the same time, in the same chamber, a shrinkage strain test was performed, according to the method in [98]. These tests allowed us to determine the above-mentioned long-term mechanical parameters of LSA concrete. Further details are provided in papers [3,5]. The loading scheme for the elastic strain and creep strain testing is presented in Figure 2 in [5].

## 3. Test Results for Mechanical Properties

### 3.1. Compressive Strength of LSA Concrete

The compressive strength test results for the LSA concrete, performed on cubical and cylindrical specimens, are presented in Figure 3 in [5]. The LC1 and LC2 LSA concrete mixtures obtained an LC 35/38 class, in accordance with [100], seven days after sample preparation (see Figure 3 in [5]). These mixtures eventually obtained two additional classes, reaching the LC 45/50 class, in accordance with [100], with a density grade of D1.8 at 28 days of age. The compressive strength tests on the LSA concrete were performed on cubic samples with dimensions of 150 × 150 × 150 mm, according to the standard in [93], while the cylindrical compressive strength tests were carried out on samples with a diameter of *d* = 94 mm and a height of *h* = 190 mm, assuming that the cross-section of the samples was the same as that intended for the creep tests (and auxiliary shrinkage tests). The given values (Figure 3 in [5]) are average values from the tests on three specimens, except for the results of the tests on the cylindrical compressive strength of the LSA concrete at the age of 28 days, which were obtained as average values from ten specimens. In this case, the strengths discussed were 49.2 MPa for the LC1 LSA concrete and 47.8 MPa for the LC2 LSA concrete, obtaining standard deviation values of 2.49 MPa and 1.21 MPa, respectively.

The mean values of compressive strength obtained from the three cube samples 28 days after their production for the LC1 and LC2 concretes were 58.3 and 57.6 MPa, respectively, and the standard deviation values were 0.48 and 0.87 MPa, respectively. More details are given in [3,5]. The expanded measurement uncertainty of the concrete cube compressive strength did not exceed the value of 1.28% for the LC1 LWAC and 1.24% for the LC2 LWAC. The results of these tests up to the 60th day of concrete curing have been previously published in articles by Rogowska and Lewiński [6,7].

In addition to compressive strength, article [5] provides test results for tensile strength, including:

- Axial tensile strength tested on cylindrical specimens, according to [94];
- Splitting tensile strength tested on cube specimens, according to [95];
- Flexural strength tested on prismatic specimens, according to [96].

### 3.2. Investigation and Analysis of Secant Elasticity Modulus of LSA Concrete

Experimental results for the secant elasticity modulus of the LSA concrete for the LC1 mixture are presented in Figure 3, and for the LC2 mixture, in Figure 4. The tests were carried out after 7, 14, 28, 60, and 120 days, and then after 300 and 400 days. In both cases, the secant elasticity modulus values increased by approximately 13% between 7 and 28 days. The results of the secant moduli of the LC1 and LC2 concretes showed a similar growth over 60 days. Previously published research results [6,7] concerned the age of concrete up to 60 days. During subsequent tests carried out after 100 days, the secant elasticity modulus values stabilized for both concretes, LC1 and LC2, at a level of approximately 23.5 GPa. Tests on the secant elasticity modulus of the LSA concrete (see Figure 5 in paper [3]) were carried out in accordance with the standard in [92] for specimens with a diameter of *d* = 94 mm and height of *h* = 190 mm, using the same specimen cross-section as those intended for the creep tests. The results obtained (see Figure 3 and Figure 4) are mean values from tests carried out on three samples, except for the secant elasticity modulus test results at 28 days of concrete age, which were obtained as average values from ten specimens. These moduli amounted to 25.15 GPa for the LC1 LSA concrete and 25.79 GPa for the LC2 LSA concrete, with standard deviations of 1135 MPa and 601 MPa, respectively. The expanded measurement uncertainty of the secant elasticity modulus did not exceed 2.03% for the LC1 LSA concrete and 1.92% for the LC2 LSA concrete.

The coefficients of variation of the elasticity modulus, which are 4.51% and 2.33%, indicate very good concrete homogeneity in this regard, both for the LC1 and LC2 concrete. It can be concluded that the measurement uncertainty of the test results is very small and, for the individual concretes, is smaller than the coefficients of variation.

The course of changes in the modulus of elasticity of the tested concrete with this type of lightweight aggregate, illustrated in Figure 3 and Figure 4, requires some commentary. Shrinkage tests carried out as part of the current research (see the next section) indicate that after 28 days, these strains had not yet reached half of their final value. However, concrete curing was completed (in accordance with generally accepted principles) 28 days after concrete casting. Reducing drying shrinkage is one of the most important goals in concrete curing. For concrete with porous aggregate, however, drying shrinkage proceeds differently than in plain concrete.

The course of this shrinkage may be influenced by the drying of water inside the aggregate due to moisture migration in the LSA concrete, and this effect increases after the curing process is completed. In this case, a growth in drying shrinkage may occur throughout the entire concrete volume. In contrast to plain concrete, the growth in strength of the cement matrix does not compensate for the increase in shrinkage stresses, because, in the zones of its contact with the porous aggregate, the matrix may dry out faster. The increase in these shrinkage stresses may, in turn, initiate the development of micro-cracks, which, although they do not significantly affect the compressive strength of lightweight aggregate concrete, may reduce both the tensile strength and the modulus of elasticity of the lightweight concrete after curing.

### 3.3. Investigation and Analysis of Shrinkage Strain in LSA Concrete

Measurements of the shrinkage strain for the LC1 and LC2 class concrete mixes were conducted using two methods: basic testing, using the Amsler standard method [97], was conducted on three prismatic samples with dimensions of 100 mm × 100 mm × 500 mm for both types of lightweight concrete, while additional shrinkage tests (carried out to determine creep strains), according to the applicable standard [98], were performed on cylindrical samples with a diameter of *d* = 94 mm and a height of *h* = 3*d* = 282 mm, assuming test samples with the same geometry as those used for the creep tests, in accordance with [94]. The Amsler method [97] tests lasted 325 days from the preparation of the LC1 concrete samples and 319 days from the preparation of the LC2 concrete samples, which was caused by a six-day difference in specimen preparation and instrumentation time. Initially, readings were taken at intervals of several days, then weekly, then monthly, and after the results had stabilized after a period of approximately 150 days, readings were taken at longer time intervals. Figure 5 presents the results of the concrete shrinkage development tests obtained by the Amsler method [97] for the LC1 and LC2 concretes and, for comparison, the analytical results (see below).

Between days 7 and 14, slight shrinkage was observed for the LC1 concrete, while the LC2 concrete showed a steady growth in shrinkage over the first 58 days. Samples of both the LC1 and LC2 lightweight concretes showed similar shrinkage increases over approximately 320 days, and similar final results were obtained for both concretes, with mean values of 0.58 and 0.61 mm/m for the LC1 and LC2 concretes, respectively, and standard deviations of 0.020 and 0.031 mm/m, respectively; thus, the shrinkage of the LC1 concrete was slightly lower than that of the LC2 concrete. The results of the tests up to 58 days of concrete age, in which shrinkage strains were measured by the standard Amsler method [97], were published earlier in [6,7]. The expanded uncertainty of shrinkage strain measurement, determined by the Amsler method, for the LSA concrete LC1 at the age of 270 and 325 days was (in percent) 5.2%, and for the LC2 concrete at the age of 270 and 319 days was 4.9%, while its value was *U* = 0.03 mm/m for both the LC1 and LC2 LSA concretes. Concrete shrinkage tests, carried out in accordance with the applicable standard EN 12390-16 [98], were started 28 days after the preparation of both types of LSA concrete samples because all cylindrical samples were cut from blocks, and were completed simultaneously with creep tests (as accompanying tests).

These tests were carried out at *t* = 1050 days from the production of specimens for the LC1 concrete, and *t* = 1044 days for the LC2 concrete, in a special laboratory chamber, where both creep and shrinkage readings were taken, and the temperature and humidity were constantly monitored and kept constant (see Figure 1 in paper [5]). For samples tested in accordance with EN 12390-16 [98], it was necessary to add to the shrinkage strain increments (measured during creep strain tests) the initial shrinkage value assumed in both cases, based on the Amsler test as 0.23 mm/m, to prepare the graphs for the total shrinkage strain (Figure 6). The average increase in shrinkage strains occurring during creep tests, determined according to the EN [98] standard, for the LC1 concrete, was 0.29 mm/m at the age of *t* = 419 days, and the average increase in shrinkage strains for the LC2 concrete at the age of *t* = 413 days was 0.45 mm/m. A comparison of the course of the concrete shrinkage strains from the tests, in accordance with the standard in [98], and the analytical results is presented in Figure 6. The results obtained for the shrinkage strains are much higher than those in [16].

### 3.4. Investigation of Creep–Recovery Strain in LSA Concrete Under Cyclic Loading

According to the ITB Instruction No. 194/98 [94], the creep measurements of concrete with lightweight sintered aggregate were performed using the LC1 and LC2 concrete mixtures, in both cases using three cylindrical specimens with the following dimensions: a diameter of *d* = 94 mm and a height of *h* = 3*d* = 282 mm, i.e., the same type of specimens used for shrinkage tests in accordance with the standard method in [98]. The LSA concrete specimens were 28 days old at first loading.

**Figure 7:**
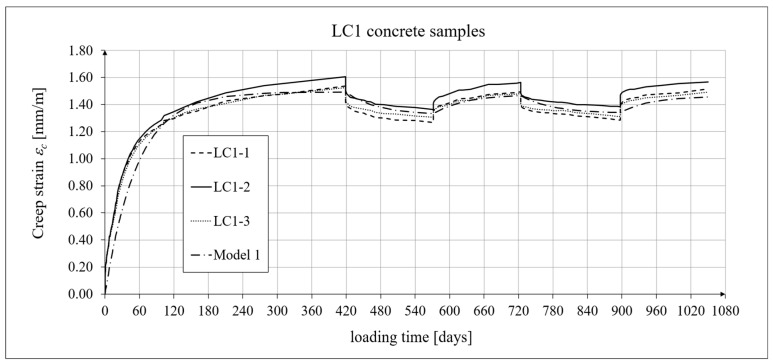
A comparison of the creep–recovery strain, according to Model 1, and experimental results for the LC1 concrete specimens.

**Figure 8:**
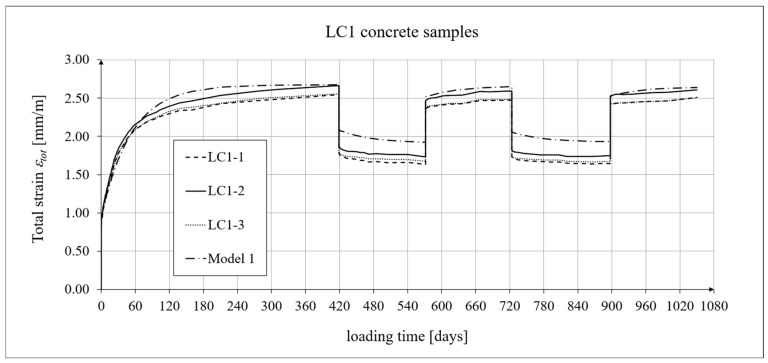
A comparison of the total strain, according to Model 1, and the experimental results for the LC1 concrete specimens.

The creep-testing machine pan loads were selected as approximately 1/3 of the concrete’s cylindrical strength, according to ITB Instruction No. 194/98 [94]. When the stress-to-strength ratio (*σ*/*f_c_*) is less than 0.4, creep is roughly linear. The LC1 concrete specimens were loaded at first (on 10 December 2019) with 110 kg weights on a balance pan, in a ratio of 100:1, resulting in stresses in the samples of 15.55 MPa, which was 0.316 times their cylindrical strength. The LC2 concrete specimens (on 16 December 2019) were loaded with 120 kg weights, resulting in stresses in the samples of 17.00 MPa, which was 0.355 times their cylindrical strength (thus yielding an average value of 0.335 for the LC1 and LC2 concrete samples). During the creep tests, the first loading period for the LC1 concrete was 419 days, and 413 days for the LC2 concrete. The duration of this loading period was selected to exceed one year (the time at which the creep coefficient is typically determined) and to achieve some stabilization of the creep results. Subsequent loading and unloading periods were 152–153 days for both concretes to achieve stable creep–recovery values. On 9 February 2021, both types of samples were unloaded to 10% of the initial loading (to 11 and 12 kg), and creep strain readings for the specimens were taken after the unloading. On 12 July 2021, the specimens were reloaded to 100% of the initial loading, and on 14 December 2021, both types of specimens were again unloaded to 10% of the initial loading. This period lasted 20 days longer due to the somewhat atypical creep–recovery patterns, especially for the LC1 concrete. On 7 June 2022, the specimens were loaded again to 100% of the initial loading, and the load was maintained until 10 November 2022. As a result of the six-day difference in loading time between the LC1 and LC2 concrete specimens (caused by the 6-day difference in their acquisition times), the creep strain test under cyclic loading was finished after 1050 days for the LC1 concrete and after 1044 days for the LC2 concrete, using the three loading and two unloading intervals described above in both cases. The creep–recovery experimental results for the LC1 concrete specimens are shown in Figure 7 and Figure 8 for the loading time *t* = 1050 days.

**Figure 9:**
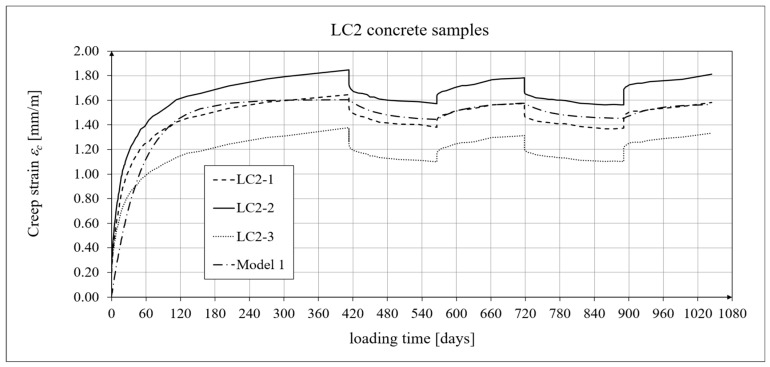
A comparison of the creep–recovery strain, according to Model 1, and experimental results for the LC2 concrete specimens.

**Figure 10:**
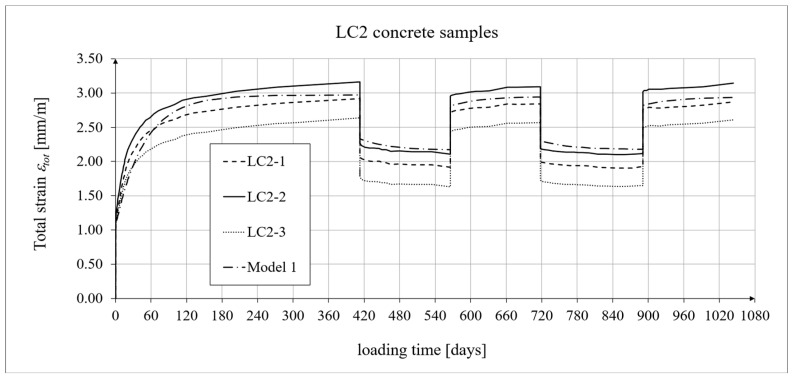
A comparison of the total strain, according to Model 1, and experimental results for the LC2 concrete specimens.

The creep–recovery experimental results for the LC2 concrete specimens are shown in Figure 9 and Figure 10 for the loading time *t* = 1044 days. The plots show the measurement results for the creep–recovery strains only, as well as the total strains of the specimens. The use of only three samples from each concrete for creep testing resulted from the adopted Instruction No. 194/98 [94] and the European standard [99] (which requires the use of two samples), as well as from equipment limitations due to the availability of two creep-testing machines, each of which can test three samples simultaneously. Therefore, each specimen strain was monitored individually, and mean values were determined solely to calculate the average creep coefficient from the three specimens for both concrete mixtures.

The basic results from long-term tests on the LC1 LSA concrete after the loading time of 419 days, along with the measurement uncertainty *U*, are given in Table 2 in [5]. The method of determining the expanded measurement uncertainty is described in detail in [3,5]. The measurement uncertainty concerns the total (*ε_tot_*), elastic (*ε_e_*), and creep strains (*ε_c_*), and the creep coefficient *φ* = *ε_c_*/*ε_e_* of the LC1 concrete after the loading time of 419 days, together with their statistical evaluation. The mean value of the creep coefficient *φ* was 2.11, with a standard deviation of 0.037. The basic results and measurement uncertainty of the total strain, elastic strain, and creep strain, and the creep coefficient of the LC2 concrete after the loading time of 413 days, are given in Table 3 in [5]. The average value of the creep coefficient *φ* was 1.94, with a standard deviation of 0.214.

In general, the creep coefficient is calculated as the ratio of the creep strain to the elastic strain (*φ* = *ε_c_*/*ε_e_*). It is determined for *t* = ∞, but in practice after a period of at least 1 year [94], as in the case under consideration, when the loading time *t* was 419 days for the LC1 concrete and 413 days for the LC2 concrete. The expanded relative measurement uncertainty (*U* [%]), which concerns the total (*ε_tot_*), elastic (*ε_e_*), and creep strains (*ε_c_*), and the creep coefficient *φ* = *ε_c_*/*ε_e_* of the LC1 concrete after the loading time of 419 days, together with their statistical evaluation, is presented in Table 2 (the maximum values are bold).

The expanded relative measurement uncertainty (*U* [%]) of the total strain, elastic strain, and creep strain, and the creep coefficient of the LC2 concrete after the loading time of 413 days, is presented in Table 3.

For rheological strain testing, the measurement uncertainties determined in this way are slightly higher than those in the case of strength tests because a different type of sensor was required for long-term strain measurements; however, these uncertainties are quite small compared to the dispersion of the rheological strain measurement results.

## 4. Models for Rheological Behavior of LSA Concrete

### 4.1. Secant Elasticity Modulus Models of LSA Concrete

The secant elasticity modulus of concrete can be determined based on its final value *E_t_*_=∞_ and the loading time, adopting the same assumptions as in the strain hereditary theory, based on the theory of a viscoelastic body. The following Arutiunian relationship for the modulus of elasticity was adopted [21,22]:

*E*(*τ*) = *E_t_*_=∞_ (1 − *β* e^−*ατ*^), (1)

where *α* and *β* are experimental constants. These constants were determined from experiments and are described below. The *α* constant has dimension [days^−1^], the *β* constant is dimensionless.

A comparison of changes in the value of the secant elasticity modulus for the LC1 concrete, based on the tests conducted in accordance with the standard in [92] and analysis using the Arutiunian model [21] (see Equation (1)), is shown in Figure 3, while a similar comparison for the LC2 concrete is shown in Figure 4.

### 4.2. Shrinkage Models for LSA Concrete

The shrinkage of concrete has been the subject of many publications. For the purposes of this publication, the Glanville model was used (ref. [101]), in which the shrinkage strain is determined according to the following formula:

*ε_cs_* (*t*) *= ε*_0_ (1 − e^−*st*^). (2)

The test results for the LC1 and LC2 concrete mixes, carried out according to the standards in [97,98], were compared to the Glanville model [101].

Figure 5 presents the results of the concrete shrinkage development tests obtained by the Amsler method [97] for the LC1 and LC2 concretes and, for comparison, obtained according to the Glanville model [101] (see Equation (2)), assuming *ε*_0_ = 0.00060 and *s* = 0.020 [days^−1^]. A comparison of changes in the values of concrete shrinkage strains in the tests, in accordance with the standard in [98] and the Glanville model [101], assuming *ε*_0_ = 0.00052 and *s* = 0.027 [days^−1^] for the LC1 concrete, and *ε*_0_ = 0.00065 and *s* = 0.026 [days^−1^] for the LC2 concrete, is shown in Figure 6.

### 4.3. Creep–Recovery Hereditary Models—Parameterization of Constitutive Relationships

This paper examines models describing rheological constitutive laws and their parameters, based directly on the theory of hereditary creep strain with aging, which determine strain development in LSA concrete with relatively new sintered aggregate. Creep strain analysis was performed using four long-term models, but the graphs only present the results obtained on the basis of Models 1 and 2, described below. Based on the studies of partially reversible creep strain in LSA concrete, procedures were developed to describe the long-term characteristics of this concrete for the following constitutive models:

1. Hereditary model with concrete aging (and its varying elasticity modulus);
2. Hereditary strain model with concrete aging (modified by Bažant [23]);
3. Hereditary elastic model;
4. Concrete aging model.

The total strain ε(t) is a superposition of strain increments for individual load values, according to the Boltzmann superposition principle [22]. Stress increments can be described as a continuous function of time if they occur at short intervals. In this case, applying the hereditary law to the uniaxial stress state [95], the superposition principle can be presented in the form of a Volterra integral equation:

(3) $$\varepsilon (t)=\int_{t_{0}}^{t}\frac{\partial \sigma (\tau )}{\partial \tau }J(t,\tau )d\tau ,$$

where the creep function (compliance function), denoted by the symbol J(t,τ), determines the proportionality of the mutual increments of strain and stress. The creep function (compliance function) takes the form [22]:

(4) $$J(t,\tau )=\frac{1}{E(\tau )}+C(t,\tau ),$$

where E(τ) is the concrete elasticity modulus, varying in time (see Equation (1)), and C(t,τ) is the creep measure described below for individual creep models.

The long-term model of strain inheritance with the aging of concrete and its changing modulus of elasticity, called the Arutiunian theory of hereditary creep strains [21,22,90,102] (hereinafter referred to as Model 1), is characterized by the creep function defined by Formulas (4) and (5). By adopting this hereditary model, the following quantification of the creep measure (creep compliance) is assumed:

(5) $$C(t,\tau )=\left(C_{0}+\frac{A_{1}}{\tau }\right)\left[\;1-e^{-\gamma (t-\tau )}\right],$$

where (*C*_0_ + *A*_1_/*τ*) is a function of concrete aging (*C*_0_ is a basic creep measure, and *A*_1_ is an aging coefficient), while the function [1 − e^−*γ*(*t*−*τ*)^] describes the development of creep over time, where *γ* is a time evolution coefficient.

The classical Volterra integral equation was modified by adding to the right side of the equation a term expressing the increase in concrete strain at time *t*, occurring after applying a stress that remains constant in time, i.e., *σ*(*t*_0_) = const at time *t*_0_, yielding the form:

(6) $$\varepsilon (t)=\sigma (t_{0})J(t,t_{0})+\int_{t_{0}}^{t}\frac{\partial \sigma (\tau )}{\partial \tau }J(t,\tau )d\tau .$$

This equation can be found in many publications (e.g., Ref. [22]) and standard documents, such as the Model Code 2010 [103], Model Code 2020 [39], and EN 1992-1-1 2023 [104]. Within the descriptions of individual models, it is possible to provide formulations of Equation (4), representing the compliance function *J*(*t*,*τ*) and taking into account the aging of concrete, the development of its creep over time, and the variable modulus of elasticity. Substituting Equation (4), the expression for the hereditary law (3) takes the form:

(7) $$\varepsilon (t)=\int_{t_{0}}^{t}\frac{\partial \sigma (\tau )}{\partial \tau }\left[\frac{1}{E(\tau )}+C(t,\tau )\right]d\tau ,$$

while after such substitution, Equation (6) takes the form:

(8) $$\varepsilon (t)=\sigma (t_{0})\left[\frac{1}{E(t_{0})}+C(t,t_{0})\right]+\int_{t_{0}}^{t}\frac{\partial \sigma (\tau )}{\partial \tau }\left[\frac{1}{E(\tau )}+C(t,\tau )\right]d\tau .$$

However, in the present consideration, unlike in publication [5], in further analysis, we will not use the integral Equation (8), but directly apply the superposition principle in the form of Equation (7). Suppose that at the age of concrete *t*_0_, a constant stress *σ*(*t*_0_) is applied, it can be described in the form of the Heaviside function [22,105]:

(9) $$\sigma (t)=\sigma (t_{0})H(t-t_{0}).$$

The derivative of the Heaviside step function *H*(*t*) is the Dirac delta function *δ*(*t*). Therefore, the partial derivative of stress over time now takes the form:

(10) $$\frac{\partial \sigma (\tau )}{\partial \tau }=\sigma (t_{0})\;\delta (\tau -t_{0}),$$

where *δ*(*t*) is the Dirac delta function. Hence, after substituting (10) into (7) and using the sifting property of the Dirac delta function, the creep strain at intervals from *t*_0_ to *t*_1_ can be described by the following equation:

(11) $$\varepsilon (t)=\sigma (t_{0})\left[\frac{1}{E(t_{0})}+C(t,t_{0})\right],$$

where

(12) $$E(t_{0})=E_{t=\infty }\left(1-\beta e^{-\alpha t_{0}}\right),$$

(13) $$C(t,t_{0})=\left(C_{0}+\frac{A_{1}}{t_{0}}\right)\left[\;1-e^{-\gamma (t-t_{0})}\right].$$

The theory of linear viscoelasticity is based on the assumptions that two linear relations apply in such a body: Hook’s law and the relation between stress and strain rate based on Newton’s law, in which the proportionality constant is a viscosity coefficient. The constitutive equation for a linearly viscoelastic body has the form of Formula (11). The assumptions of the theory of hereditary creep strain are more general, because the total strain is a superposition of strain increments for individual loads and can be represented by the integral Equation (3). This integral equation can be applied to a series of constant stress increments and decrements, particularly under cyclic loading conditions, and can be described mathematically as follows.

Suppose that at time *t*_0_, a stress *σ*(*t*_0_) is applied, which acts until time *t*_1_, when a stress *σ*(*t*_1_) is applied, which, in turn, acts until time *t*_2_, when a stress *σ*(*t*_2_) is applied. The stress change pattern in individual intervals can also be described using the Heaviside function:

(14) $$\sigma (t)=\sigma (t_{0})\;\left[H(t-t_{0})-H(t-t_{1})\right]+\sigma (t_{1})\left[H(t-t_{1})-H(t-t_{2})\right]+\sigma (t_{2})\;\left[H(t-t_{2})\right].$$

The partial derivative of the function *σ*(*τ*), with respect to *τ*, now takes the form:

(15) $$\frac{\partial \sigma (\tau )}{\partial \tau }=\sigma (t_{0})\;\delta (\tau -t_{0})+\left(\sigma (t_{1})\;-\sigma (t_{0})\;\right)\;\;\delta (\tau -t_{1})+\left(\sigma (t_{2})\;-\sigma (t_{1})\right)\;\delta (\tau -t_{2}).$$

Therefore, after substituting (15) into (7) and using the sifting property of the Dirac delta function, the following equation for the creep–recovery strains in the range from *t*_2_ to *t*_3_ can be obtained:

(16) $$\varepsilon (t)=\sigma (t_{0})\left[\frac{1}{E(t_{0})}+C(t,t_{0})\right]+\left(\sigma (t_{1})-\sigma (t_{0})\right)\left[\frac{1}{E(t_{1})}+C(t,t_{1})\right]+\left(\sigma (t_{2})\;-\sigma (t_{1})\right)\left[\frac{1}{E(t_{2})}+C(t,t_{2})\right],$$

where *E*(*t*_0_) and *C*(*t*, *t*_0_) are defined by Equations (12) and (13), while *E*(*t*_1_), *C*(*t*, *t*_1_), *E*(*t*_2_), and *C*(*t*, *t*_2_) are defined as follows:

(17) $$E(t_{1})=E_{t=\infty }\left(1-\beta e^{-\alpha t_{1}}\right),$$

(18) $$C(t,t_{1})=\left(C_{0}+\frac{A_{1}}{t_{1}}\right)\left[\;1-e^{-\gamma (t-t_{1})}\right],$$

(19) $$E(t_{2})=E_{t=\infty }\left(1-\beta e^{-\alpha t_{2}}\right),$$

(20) $$C(t,t_{2})=\left(C_{0}+\frac{A_{1}}{t_{2}}\right)\left[\;1-e^{-\gamma (t-t_{2})}\right].$$

Then, proceeding recursively, for the creep–recovery strains in the range from *t_n_* to *t_n_*_+1_, the following relationship can be derived from Equation (7):

(21) $$\varepsilon (t)=\sigma (t_{0})\left[\frac{1}{E(t_{0})}+C(t,t_{0})\right]+\sum_{i=1}^{n}\left(\sigma (t_{i})-\sigma (t_{i-1})\right)\left[\frac{1}{E(t_{i})}+C(t,t_{i})\right]$$

where *E*(*t*_0_) and *C*(*t*, *t*_0_) are defined by the Equations (12) and (13), while *E*(*t_i_*) and *C*(*t*, *t_i_*) are defined as follows:

(22) $$E(t_{i})=E_{t=\infty }\left(1-\beta e^{-\alpha t_{i}}\right),$$

(23) $$C(t,t_{i})=\left(C_{0}+\frac{A_{1}}{t_{i}}\right)\left[\;1-e^{-\gamma (t-t_{i})}\right],$$

and the limit value of the creep measure are

(24) $$C(\infty ,\tau )=\left(C_{0}+\frac{A_{1}}{\tau }\right).$$

Bažant and Jirásek (see Refs. [22,102]) obtained a similar mathematical model, defined as the Age Adjusted Effective Modulus (AAEM). The need to use the integral equation in the form (6), i.e., the need to distinguish the initial term, arose from the lack of a suitable initial condition. The description of the strain variation presented in Equation (21) can be obtained by applying the appropriate condition and utilizing the Heaviside function. Therefore, if the initial condition is correctly formulated, correction of the Boltzmann superposition principle is unnecessary, and modifying the integral equation to the form of Equation (6) is not necessary.

In the present paper, four rheological constitutive models were analyzed for comparative purposes.

- Model 1 is the Arutiunian theory of hereditary creep strains with concrete aging and the varying elasticity modulus [21,22,91,102], which is the hereditary model described above (see Equation (5)).
- Model 2 is a model of hereditary creep strains with aging (and the varying elasticity modulus of concrete), modified by Bažant [23], in which the creep measure has the following form:

(25) $$C(t,\tau )=\left(C_{0}+Ae^{-\gamma \tau }\right)\;\left(1-e^{-\gamma (t-\tau )}\right).$$

- Model 3 is an “elastic inheritance” model [89,90], in which the creep measure does not depend on the aging coefficient *A*_1_, which is assumed to be equal to zero in Equation (5).
- Model 4 is a well-known concrete aging model [89,90], in which the following creep measure is assumed in the interval from *t*_0_ to *t*_1_:

(26) $$C(t,t_{0})=C(t)-\;C(t_{0}),$$

where

(27) $$C(t)=\varphi_{0}\left(\infty \right)\;\left(1-e^{-b_{0}t}\right)/E\left(t_{0}\right),$$

(28) $$C(t_{0})=\varphi_{0}\left(\infty \right)\;\left(1-e^{-b_{0}t_{0}}\right)/E\left(t_{0}\right).$$

Similarly at subsequent time intervals, until all the material parameters necessary for calculating the elastic and creep strains required in Equation (21) have been determined.

### 4.4. Comparison of Constitutive Rheological Models for Creep–Recovery Strain with Test Results

The analysis of creep strain was performed with respect to cyclic loadings, taking into account the rheological model of hereditary creep strains (3) and the following four long-term models, according to: the Arutiunian theory of hereditary creep strains with aging (refs. [21,22,90,102]), the modified hereditary theory with Bažant aging function [23], the “elastic inheritance” theory and the aging theory [89,90]. The LSA concrete specimens were first charged on the 28th day. Measurements of elastic, total, and shrinkage strains for the LC1 and LC2 concrete samples were carried out using the following loading program to determine creep strains:

- In the case of samples made of the LC1 concrete, the first loading phase lasted from day 1 to 419 at a stress of 15.55 MPa, the creep–recovery strains were recorded during the first unloading phase from days 419 to 572 at a stress of 1.56 MPa, the first reloading phase lasted from days 572 to 724 at a stress of 15.55 MPa, the next creep–recovery strains were measured during the second unloading phase from days 724 to 897 at a stress of 1.56 MPa, and the second reloading phase lasted from days 897 to 1050 at a stress of 15.55 MPa.
- In the case of the samples made from the LC2 concrete, the first loading phase lasted from day 1 to 413 at a stress of 16.96 MPa, the creep–recovery strains were measured during the first unloading phase from days 413 to 566 at a stress of 1.70 MPa, the first reloading phase lasted from days 566 to 718 at a stress of 16.96 MPa, the next creep–recovery strains were measured during the second unloading phase from days 718 to 891 at a stress of 1.70 MPa, and the second reloading phase lasted from days 891 to 1044 at a stress of 16.96 MPa.

For comparative calculations of long-term strains, the number of days defining the time intervals was calculated based on the concrete age at the time of sample loading, which was 28 days. Therefore, for the LC1 concrete samples: *t*_0_ = 28 days, *t*_1_ = 419 + 28 = 447 days, and similarly the following values, *t*_2_ = 600 days, *t*_3_ = 752 days, and *t*_4_ = 925 days. Likewise, for the LC2 concrete samples: *t*_0_ = 28 days, *t*_1_ = 441 days, *t*_2_ = 594 days, *t*_3_ = 746 days, and *t*_4_ = 919 days.

The Arutiunian model of the modulus of elasticity is represented by Formulas (12), (17), etc., in which the following data are adopted: *α* = 0.14 [days^−1^], *β* = 0.35, and the values of the final elastic moduli (determined after 400 days after concreting) are *E_t_*_=∞_ = 23.62 GPa for the LC1 concrete (see Figure 3) and *E_t_*_=∞_ = 23.87 GPa for the LC2 concrete (see Figure 4). The Glanville concrete shrinkage model is represented by Formula (2), where *ε*_0_ = 0.00052 and *s* = 0.027 [days^−1^] for the LC1 concrete and *ε*_0_ = 0.00065 and *s* = 0.026 [days^−1^] for the LC2 concrete (see Figure 6) to evaluate the additional shrinkage (*ε_as_*) after 28 days of pouring the concrete. The Arutiunian theory of hereditary creep strains with concrete aging and the varying elasticity modulus (Model 1) requires quantification of the creep measure parameters represented by Formulas (7), (13), (18), etc., and the following values were assumed based on recommendations in [90] and a short iteration procedure.

The procedure for calibrating the model parameters is very simple because, unlike the standard model, both models of hereditary creep strain require only three parameters: the basic creep measure *C*_0_, the aging coefficient *A*_1_ (or *A*), and the time evolution coefficient *γ*. The parameter *γ* is typically assumed to be between 0.018 and 0.03, allowing its value to be initially determined in several iterative steps. The value of the coefficient *A*_1_ was initially assumed based on [90], although other literature sources can also be used. The basic creep measure *C*_0_ was calculated by comparing the analytical results with the test results. The subsequent steps of the calibration procedure were as follows:

- Adopting iterative steps for the coefficients *C*_0_, *A*_1_, and *γ*;
- Initiating forward and backward iterations for these coefficients;
- Comparing the experimental results with the analytical models by calculating the root mean square errors (RMSEs);
- Selecting the variant when the RMSE reaches the minimum value for all coefficients.

Table 4 presents the values of the root mean square error (RMSE) for the two concrete creep models of the two lightweight concretes, LC1 and LC2, for individual time intervals from the moment of loading. The number *n* indicates the number of squared differences used to determine the error.

In the case of the LC2 concrete samples, the differences between the model and test results are greater than those for the LC1 concrete due to the greater scatter of the test results themselves, which was mainly caused by the higher W/C ratio. The analysis omitted the first loading period up to 48 days for the LC1 concrete and 42 days for the LC2 concrete. In this interval, assessing the modeling accuracy is unreliable because the creep curves are nearly vertical and undergo significant changes from zero to near-final values.

For the LC1 concrete, the coefficient *C*_0_ = 6.632 · 10^−6^ [MPa^−1^], the aging coefficient *A*_1_ = 0.002504 [MPa^−1^·days], *γ* = 0.018 [days^−1^], and for the LC2 concrete, the coefficient *C*_0_ = 5.338 · 10^−6^ [MPa^−1^], the aging coefficient *A*_1_ = 0.002492 [MPa^−1^·days], and *γ* = 0.02 [days^−1^]. Moreover, using the stress values given above, the elastic and creep strains for individual time intervals were determined according to Formula (21). Similarly, creep strains were determined for Model 2 as described above, assuming for the LC1 concrete, the coefficient *C*_0_ = 1.892 · 10^−5^ [MPa^−1^], the aging coefficient *A* = 1.304 · 10^−4^ [MPa^−1^], *γ* = 0.018 [days^−1^], and for the LC2 concrete, the coefficient *C*_0_ = 2.527 · 10^−5^ [MPa^−1^], the coefficient *A* = 1.215 · 10^−4^ [MPa^−1^], and *γ* = 0.02 [days^−1^].

**Figure 11:**
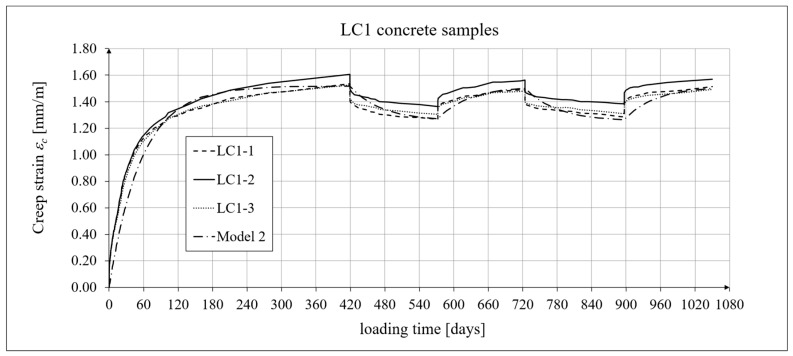
A comparison of the creep–recovery strain, according to Model 2, and experimental results for the LC1 concrete specimens.

**Figure 12:**
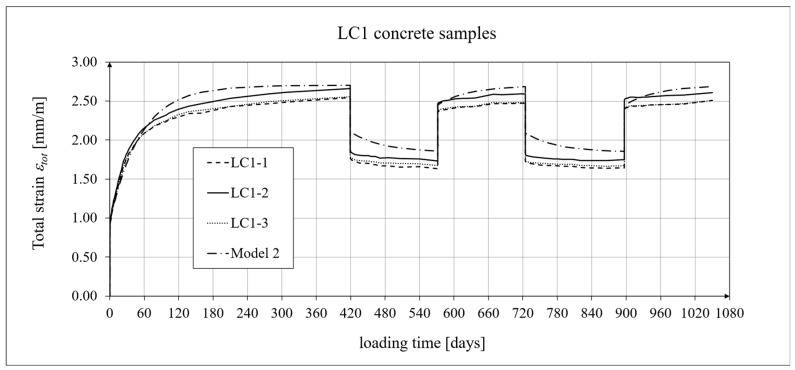
A comparison of the total strain, according to Model 2, and experimental results for the LC1 concrete specimens.

**Figure 13:**
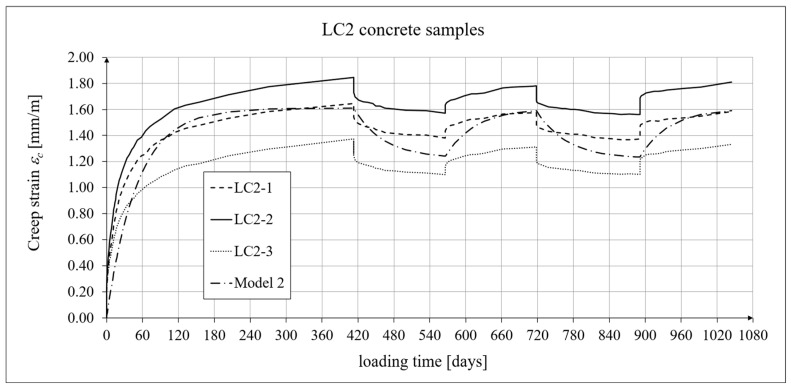
A comparison of the creep–recovery strain, according to Model 2, and experimental results for the LC2 concrete specimens.

**Figure 14:**
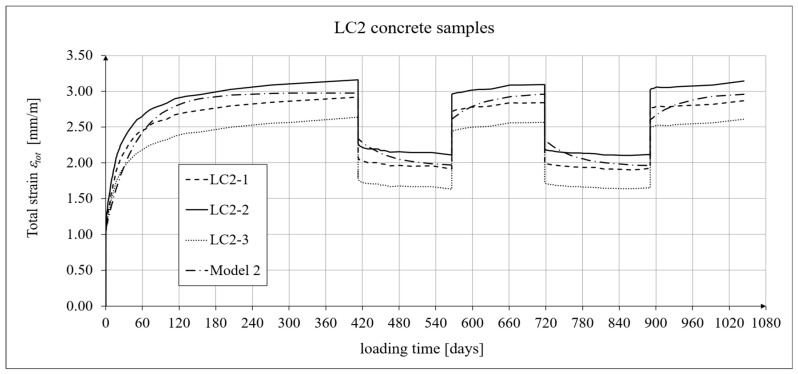
A comparison of the total strain, according to Model 2, and experimental results for the LC2 concrete specimens.

The selection of material constants was performed as follows. To correctly model the development of creep strains, the initial values of material constants were adopted based on recommendations in [90]. However, they were verified according to the recommendations of the European standard [104], using the appropriate correction factors, which are determined according to the principle that the sum of squares of differences between the model evaluation and test results is minimized. Using Equation (21), the elastic and creep strains were determined for individual time intervals, and using Equation (2), the shrinkage strain was determined. A comparison of the experimental results of the creep–recovery strain and the total strain in the LC1 concrete with the calculation results for the hereditary model (Model 1) is shown in Figure 7 and Figure 8, and for the Bažant model (Model 2) in Figure 11 and Figure 12. Similarly, a comparison of the creep–recovery strain and the total strain test results for the LC2 concrete with the results for the above-mentioned models is shown in Figure 9 and Figure 10 (Model 1) and Figure 13 and Figure 14 (Model 2). For the above models (Models 1 and 2), it is relatively easy to obtain quantitatively and qualitatively correct (and quite similar) results. For Model 3 of “elastic inheritance”, assuming the value of the coefficient *C*_0_ (see Equation (5)) as independent of time and equal to *C*(∞, *τ*), according to Equation (24), and the aging coefficient *A*_1_ = 0, the calculation results for the first loading phase are the same as in Model 1. However, in the subsequent time intervals with unloading, the stress drops in this case are significantly overestimated. In Model 4, in the model of concrete aging, according to Equations (26)–(28), upon sudden unloading, no recovery of creep strain occurs; thus, this theory represents irreversible creep strains. The analysis shows that Models 3 and 4 lead to qualitatively incorrect results, disqualifying them for cyclic loads acting on the considered lightweight concrete. The graphs below show the test and analysis results for creep–recovery strain only, as well as the total strain of the samples. The first four graphs (Figure 7, Figure 8, Figure 9 and Figure 10) compare the results for Model 1, and the next four (Figure 11, Figure 12, Figure 13 and Figure 14) compare the results for Model 2.

## 5. Discussion

Rheological tests on samples made of two lightweight concrete mixtures, aimed at determining their strains under the influence of cyclic loads, were carried out as part of research on the mechanical properties of LSA concrete with aggregate derived from fly ash. The research was carried out for two types of concrete mixtures, with a W/C ratio of 0.4 for the LC1 mix and 0.5 for the LC2 mix, which were obtained by slight modification of the mix compositions (see Refs. [12,13,16]).

The average influence of the W/C ratio was different for different tests:

- The cube compressive strength of the LC1 concrete turned out to be 2−9% higher than that of the LC2 concrete, depending on the age of the concrete, while the cylindrical compressive strength was higher by approximately 2.5%.
- The secant modulus of elasticity did not show significant differences for both concretes.
- In the Amsler shrinkage tests, the shrinkage values of the LC2 concrete were higher than those of the LC1 concrete in a time-varying manner, stabilizing after 150 days at a level of 5% higher, while in the shrinkage tests, performed according to the European Standard on cylindrical samples, the shrinkage values were also higher, but stabilized after 120 days at a level of 20% higher.
- In the creep tests on cylindrical samples, the mean values of the creep strains for both concretes were at the same level, but in the case of the LC2 concrete samples, the scatter of the results was much higher than in the case of the LC1 concrete samples.

The charts prepared for approximately the first 420 days of aging of this lightweight aggregate concrete show the development of the compressive strength and the secant elasticity moduli of both types of concrete over time. In the present work, the test results include measurements from the entire testing process, which lasted 1050 days from the moment of making the LC1 concrete samples and 1044 days for the LC2 concrete, including cyclic loading. According to the tests, the cube compressive strength of the LSA concrete in question was 57–58 MPa after 28 days. The samples made from the LC1 concrete mix achieved slightly greater strength and less shrinkage, as expected, compared to the samples made from the LC2 mix. Compared to plain concrete with similar properties, the secant elasticity modulus of the LSA concrete obtained in the tests, which was approximately 25 GPa after 28 days, has a slightly lower value, but the negative impact of such a modulus value is compensated by the lower value of the bulk density of LWAC in question. Compared to the results presented in [12,13,16], the short-term properties of the tested concretes do not differ significantly, which was confirmed by the authors’ previous research [3,5,6,7].

However, significant differences were obtained for the long-term mechanical properties. The values of shrinkage strains obtained during tests for a concrete age of approximately 116 days are almost twice as high for both the LC1 and LC2 concretes as in [12,13,16]. The creep coefficient of the LSA concrete typically ranges from 0.5 to 2.1, depending on the concrete mix (including the type of aggregate), the loading level, and the age of the concrete. The experimentally determined average final creep coefficient (see Section 3.4) obtained for the three LC1 concrete samples was approximately 2.1 for the loading time of 419 days, and for the three LC2 concrete samples, it was approximately 1.9 for 413 days, which means that the LWAC creep strains are close to the values for plain concrete. The mean value of the creep coefficient for both concretes obtained experimentally in [16] was approximately 0.60, which differs not only from the authors’ research results but also from the predictions of the standard model [100]. The results of our research differ significantly because the tests described in [16] were carried out at a lower stress level and were not conducted in creep-testing machines, with the stress in concrete being induced by prestressing forces. The creep coefficient values obtained by the authors for the age of concrete of about 116 days for both the LC1 and LC2 concrete are about three times bigger than those given in the cited works [12,13,16], but they do not differ from the values characteristic of plain concrete, which is in accordance with the standard in [100].

The important factors influencing creep are pre-wetting aggregates and aggregate type. Using pre-wetted aggregates can help decrease creep by providing internal curing, which reduces microcracking, whereas air-dried aggregates can increase specific creep. The sintered fly ash aggregates, often used in lightweight structural concrete, tend to exhibit slightly lower creep than normal concrete, providing good, long-term performance for prestressed structures.

In research on cyclic loadings, creep strain analysis was conducted, taking into account the integral hereditary law (7) and the following rheological models: the Arutiunian theory of hereditary creep strains with aging [21,22,90,102], the modified hereditary theory with Bažant aging function [23], the theory of “elastic inheritance”, and the well-known aging theory. The results obtained based on the hereditary model (Model 1) and the Bažant model (Model 2) proved to be quantitatively and qualitatively correct (and quite similar). For Model 3 (“elastic inheritance”), in the first phase of loading (without unloading), the analytical results are the same as for Model 1; however, for subsequent time intervals with unloading, the stress drops caused by unloading are significantly overestimated, according to the tests. For Model 4 (“aging of concrete”), no creep strain is recovered upon sudden unloading, so this theory models irreversible creep strains. Such behavior of Models 3 and 4 leads to qualitatively incorrect results and disqualifies them for cyclic loads acting on the considered lightweight concrete. The hereditary model with concrete aging can be applied to both plain and lightweight concrete. The creep model used is limited to linear creep when the stress-to-strength ratio (*σ*(*t*_0_)/*f_cm_*(*t*_0_)) is less than 0.4. For high stress levels, creep is no longer proportional to the applied stress. This non-linearity of creep may be considered by adjusting the creep coefficient.

Experimental creep–recovery tests investigated whether ratcheting occurred. However, this phenomenon was not detected, primarily due to the slight plastic strain values in the LWAC. Therefore, cyclic loading did not increase the creep strain. The behavior of LSA concrete under cyclic loading indicates a significant increase in the aging factor in the expression of the creep measure compared to plain concrete.

Furthermore, the analysis of cyclic loading shows that the commonly used corrections to the Boltzmann–Volterra superposition principle (3) are not necessary (provided that the initial condition is correctly formulated). However, the integral equation of strain inheritance in the form of Equation (6) is cited in many publications, among others in the Model Code 2020 [39] and EN 1992-1-1 2023 [104]. Therefore, integral equations of Equation (6) or (8) can be used in a shortened form.

## 6. Conclusions

The following conclusions can be drawn from the tests and analyses carried out.

### 6.1. Conclusions Regarding the Obtained Test Results

- Based on the LSA concrete tests, it was determined that the mean final creep coefficient of the LC1 concrete samples tested after more than one year was approximately 2.1, and for the LC2 concrete samples, approximately 1.9, yielding an average value of 2.0, similar to the value for plain concrete of the same strength.
- The mean recovery value for the LC1 lightweight concrete was determined by comparing the creep strains at the end of the first loading period and the first unloading period, obtaining 15.62%, and for the LC2 concrete, 16.75%, i.e., on average, an approximation 16.2%, which is less than that for plain concrete of the same strength.
- For creep (and accompanying shrinkage), in the tests on the LC1 concrete and LC2 concrete, three samples were used in each case due to the requirements of the instructions in [94] and the requirements of the European standard [99], as well as limited access to creep testing machines.
- No ratcheting phenomenon was observed (probably due to minor plastic deformations), and therefore, cyclic load changes did not increase the creep deformation of the tested concrete.
- Considering the properties of LSA concrete mixtures, further research is planned using the addition of fibers, which should increase the tensile strength for concrete, elasticity modulus, and frost resistance.

### 6.2. Conclusions Regarding the Model Analysis Results

- Standard models cannot be applied directly and require additional calibration using correction coefficients. However, these models are very complex compared to those based on the theory of hereditary creep strain.
- Since the use of standard models does not improve the approximation of experimental results without additional calibration, the authors suggest reconsidering the direct application of basic hereditary models for LSA concrete.
- The application of four long-term models was analyzed. Of these models, the Arutiunian theory of hereditary creep strain with aging and the modified hereditary theory with Bažant aging function yielded quantitatively and qualitatively correct results.
- There is no need to correct the Boltzmann superposition principle given in the form of a Volterra integral Equation (3) as long as the initial condition is formulated correctly.

### 6.3. Conclusions for Structural Applications

- Evaluating the properties of LSA concrete under long-term cyclic loading requires experimental testing, and standard data alone are insufficient. This also results from the need to apply correction factors in accordance with current standards.
- Knowledge of the properties of LSA concrete and useful long-term models, including those based on the theory of hereditary creep, is essential for the design of prestressed structures made of lightweight aggregate concrete subjected to time-varying loads.
- There are no contraindications to using the type of LSA concrete under consideration in structures subjected to long-term cyclic loading.
- The concrete mixtures described in the paper are suitable for use in prestressed structures subjected to long-term cyclic loading, which should contribute to benefits in the field of sustainable construction.
- The advantages include the possibility of using LSA concrete for the construction of prestressed slabs, because despite the lower modulus of elasticity than in the case of plain concrete, the lower density of LSA concrete ensures smaller deflections of the floor slabs. As the ratcheting phenomenon was not observed (probably due to minor plastic deformations), the cyclic load changes did not increase the creep deformation of the tested concrete and the deflection of the floor slabs.
- The disadvantages of the tested LSA concrete include its brittleness, which limits its applications in the case of structures in which the tensile strength of the concrete is important, e.g., in the case of significant shearing or punching.
- In a situation of decreasing access to natural crushed aggregates, reusing waste materials is now an environmental priority. As a result, the reuse of ashes for the production of concrete aggregate may, in the future, reduce the mining of raw materials.

## Figures and Tables

**Table 1:** Concrete mix compositions, test types, and number of specimens.

Components and Tests	LC1 Mix	LC2 Mix
Component	Dosage [kg/m^3^]	
Cement CEM I 42.5 N	409	419
Lightweight sintered aggregate *Certyd* 4/10	775	802
Sand	682	703
Water	164	209
Admixture *SKY 686*	3.7	3.8
Admixture *BV 18*	3.7	3.8
Test types	Number of specimens	
Cube compressive strength	18	18
Cylinder compressive strength	25	25
Secant modulus of elasticity	34	34
Tensile splitting strength	18	18
Flexural strength	18	18
Amsler shrinkage tests	3	3
Shrinkage tests acc. to CEN standard	3	3
Creep tests	3	3

**Table 2:** The expanded measurement uncertainty (*U*) of the LC1 concrete test results after 419 days.

Sample No.	*U* (*ε_e_*)	*U* (*ε_c_*)	*U* (*ε_tot_*)	*U* (*φ*)
	[%]	[%]	[%]	[%]
LC1-1	**5.56** ^1^	4.55	**2.75**	**8.42**
LC1-2	5.26	4.35	2.63	8.02
LC1-3	5.41	**4.58**	2.73	8.22
Average value	5.41	4.49	2.70	8.22
Standard deviation	0.146	0.124	0.063	0.195

^1^ The maximum values are in bold.

**Table 3:** The expanded measurement uncertainty (*U*) of the LC2 concrete test results after 413 days.

Sample No.	*U* (*ε_e_*)	*U* (*ε_c_*)	*U* (*ε_tot_*)	*U* (*φ*)
	[%]	[%]	[%]	[%]
LC2-1	4.88	4.24	2.40	6.96
LC2-2	4.60	3.80	2.53	6.62
LC2-3	**4.94** ^1^	**5.07**	**2.65**	**7.63**
Average value	4.80	4.37	2.53	7.07
Standard deviation	0.182	0.644	0.127	0.515

^1^ The maximum values are in bold.

**Table 4:** Root mean square error (RMSE) values for LWAC creep modeling.

Samples of Concrete	Loading Time		*n*	Model 1	Model 2
	From [Day]	To [Day]	[-]	[%]	[%]
LC1 concrete	48	419	16	6.92	6.24
	419	572	17	3.20	3.81
	572	724	16	3.13	5.22
	724	897	23	2.00	4.23
	897	1050	16	5.33	8.38
LC2 concrete	42	413	16	9.43	9.69
	413	566	17	15.05	10.92
	566	718	16	9.13	5.97
	718	891	23	16.36	10.98
	891	1044	16	7.55	6.01

## Data Availability

The original contributions presented in this study are included in the article. Further inquiries can be directed to the corresponding author.

## Abbreviations

The following abbreviations are used in this manuscript:

ITB	Instytut Techniki Budowlanej (Building Research Institute)
LSA	Lightweight Sintered Aggregate
LWAC	Lightweight Aggregate Concrete
LSA Concrete	Sintered Fly Ash Concrete
IMiKB	Instytut Materiałów i Konstrukcji Budowlanych (Inst. of Building Mater. & Struct.)
PC struct.	Prestressed concrete structures
W/C	Water/Cement Ratio
LC	Lightweight Concrete
CEM	Cement Class
CEB-FIP	the merger of CEB and FIP
CEB	Euro-International Committee for Concrete
FIP	International Federation for Prestressing
MC 2010	Model Code 2010
MC 2020	Model Code 2020
